# Multi-system Mechanisms in the Anti-osteoporotic Effects of Herbal Natural Products

**DOI:** 10.2174/0118715303447831260510204800

**Published:** 2026-05-21

**Authors:** Xiaoyun Li, Tongying Chen, Yunjie Zeng, Yangbo Li, Shuhua Liu, Xijian Guo, Chenhaoyu Liu, Lei Wan, Ying Li, Hongxing Huang

**Affiliations:** 1 Guangzhou University of Chinese Medicine, Guangzhou, Guangdong, China;; 2 The Third Affiliated Hospital of Guangzhou University of Chinese Medicine, Guangzhou, Guangdong, China

**Keywords:** Chinese medicine, osteoporosis, plant-based natural products, PI3K/AKT pathways, MAPK pathways, synergistic effects

## Abstract

**Introduction:**

Osteoporosis (OP) is increasingly recognized as a multi-system disorder beyond traditional hormonal and nutritional perspectives. This review aims to systematically present the therapeutic potential of natural components from Chinese medicine and herbal formulae, clarifying their mechanisms of action within human physiological systems.

**Methods:**

We conducted a comprehensive search of relevant studies in PubMed, Web of Science, CNKI, and other databases. The focus was on the use of herbal monomers and classical formulas in the field of OP. This analysis focused on their multisystem interactions and their potential molecular pathways.

**Results:**

Our analyses indicate that these natural products not only directly regulate bone metabolism through the Wnt/β-catenin, BMP/Smad, OPG/RANK/RANKL, and PI3K/AKT pathways, but they also exert anti-osteoporotic effects through multi-system regulatory mechanisms. Specifically, they exert therapeutic effects by: alleviating endocrine imbalances through regulating parathyroid hormone and estrogen levels; alleviating chronic inflammation *via* the NF-κB and MAPK pathways; regulating the composition of intestinal flora; and affecting nervous system signalling *via* sympathetic modulation. The herbal formulae particularly show synergistic effects when acting on multiple systems at the same time.

**Discussion:**

The synthesis of evidence reveals that TCM counteracts OP *via* a holistic, system-restorative mechanism. By concurrently modulating endocrine, inflammatory, intestinal, and neural functions, this multi-system strategy addresses the complex etiology of OP and presents a complementary paradigm to conventional target therapies.

**Conclusion:**

Chinese medicine offers a multi-targeted treatment option for OP through restoring balance to the physiological system. This holistic treatment strategy, derived from TCM theory and increasingly validated by modern research, offers a promising complementary therapy for the treatment of OP. It can complement traditional single-targeted therapies.

## 1 INTRODUCTION

Osteoporosis(OP), known as the “silent epidemic”, is a systemic bone disease. It is characterised by a decrease in bone mass and deterioration of the microstructure of bone tissue, which leads directly to increased bone fragility and a higher risk of fracture [1]. Global epidemiological data indicate that OP affects over 200 million people. Traditional perspectives attribute this condition to the combined effects of multiple factors, with hormonal changes such as estrogen deficiency and deficiencies in calcium and vitamin D being regarded as key risk factors [2-4]. However, as research has progressed, OP has increasingly come to be regarded as a multisystemic disorder closely linked to the endocrine, immune, reproductive, digestive, and nervous systems. This new understanding could help improve future strategies, as current treatments for OP are often unsatisfactory. Traditional Chinese medicine(TCM) formulas have the advantage and characteristics of fewer side effects and better efficacy. This is due to the fact that they are derived from natural plant products and are based on a unique therapeutic philosophy of treating people as a whole [5]. Take OP as an example, TCM posits that bones receive nourishment from the spleen and kidney systems. Therefore, OP was regarded as the outcome of energy deficiency in the kidney and spleen. Numerous Chinese herbal medicines and formulas have shown efficacy in treating OP. However, their function across multiple systems hasn’t been elucidated; this paper aims to explore their multi-system mechanisms of action [6].

## 2 MECHANISM OF OSTEOPOROSIS

Bone metabolism is so active that bone regeneration and bone remodeling occur throughout life. During the procedure, osteoblasts regulate the regeneration of bone while osteoclasts mediate bone resorption. Besides, Bone Marrow-Derived Mesenchymal Stem Cell (BMSC) plays a vital role in bone formation and regulates the function of osteoclasts *via* paracrine signaling. However, once the equilibrium between these two physiological processes breaks, especially when the osteoclast activity exceeds osteoblast regeneration, there is a high risk of osteoporosis. This leads to a decrease in bone mineral density(BMD) and a deterioration of the bone tissue.

At the molecular level, several signaling pathways, genetic elements, and modulatory molecules have been demonstrated to play important roles in the occurrence of OP. For example, the Wnt/β-catenin pathway plays an important role in regulating osteogenic differentiation and promoting bone formation. Wnt signaling orchestrates bone homeostasis, coordinately stimulating osteoblast-mediated bone formation and inhibiting osteoclast-driven bone resorption. Inhibition of Wnt antagonists, such as sclerostin (SOST) and DKK1, stimulates bone formation and increases bone mineral density. The bone morphogenetic protein (BMP)/mothers against decapentaplegic homolog(Smad) pathway also promotes osteogenic differentiation, and BMP is an important player in bone formation, inducing the differentiation of pluripotent BMSCs into osteoblasts [7, 8]. BMPs bind to receptors and recruit Smad1, Smad5, and Smad8, which are then translocated to the nucleus to regulate downstream gene expression. The osteoprotegerin(OPG)/receptor activator of nuclear factor-κB ligand (RANKL)/receptor activator of nuclear factor-κB (RANK) signaling axis serves as a core regulatory system that maintains bone homeostasis by coordinately controlling the balance between osteoclast and osteoblast activity [9]. Both OPG and RANKL can be produced by osteoblasts, and RANKL signalling promotes bone resorption by driving the maturation of osteoclasts through RANK expressed on them, whereas OPG can also bind to RANK and compete with RANKL to inhibit osteoclast-induced bone resorption and inhibit bone resorption [10]. The Hedgehog Signalling Pathway (HH) is key to regulating bone development and homeostasis *in vivo*. HH signalling is not only involved in the development of osteoclasts, but also in the development of the bone marrow. HH signaling pathway promotes the osteogenic differentiation of BMSCs by upregulating key transcription factors, including Runt-related transcription factor 2 (RUNX2) and Osterix (OSX) [11]. Two of these essential transcription factors, RUNX2 and OSX, are responsible for mediating and enabling the process of osteoblast differentiation. RUNX2 was the first defined osteoblast-specific transcription factor, while the zinc-finger protein OSX acts as its downstream effector, also important for osteoblast differentiation [12]. The p38 mitogen-activated protein kinase (p38-MAPK) pathway, *via* the Runx2 phosphorylation, exerts a differentiation-promoting effect on osteoblasts. In the context of human periosteal-derived cells(PDCs), the c-Jun N-terminal kinase(JNK) pathway emerges as a key regulator controlling the process of osteoblast differentiation [13, 14]. And molecules such as 1,25 dihydroxyvitamin D, parathyroid hormone (PTH), glucagon-like peptide-1(GLP-1), and insulin-like growth factor(IGF) all contribute to bone formation [15, 16]. All of the above mechanisms of regulation of bone metabolism by various factors can be the basis for our research and development of anti-OP drugs.

## 3 DIFFERENT SYSTEMS AND OP

Traditionally, the pathological mechanisms of OP have emphasised endocrine actions. The most distinctive one is PTH, which can regulate the Ca2+ homeostasis. The administration of PTH enhances bone formation by altering transcription in diverse pathways aimed at osteoblasts; the most dominant of these pathways are adenylate cyclase and PKA [17]. Then they target the Wnt/β-catenin pathway, the classic pathway in osteoblasts that influences bone formation [17]. In addition, glucose is an important source of bone physical processes and therefore plays an essential role in skeletal homeostasis. It has been shown that the glucose transporters (Glut1) prevent the degradation of osteoblast differentiation Runx2 by inhibiting AMPK [18]. As for the osteoclast, activation of LDH leads to the production of RANKL created by osteoclasts, which in turn stimulates NFATc1 and further drives osteoclast formation [19]. Recently, more studies indicate that inflammation is also a contributor to OP. Clinically, elevated inflammatory factors such as tumour necrosis factor (TNF) and interleukin-6(IL6) are strongly associated with an increased risk of hip and vertebral fractures in older women and men [20, 21]. Mechanistically, immune cells play a complex role in this process: Activated T cells promote osteogenic differentiation of BMSCs by secreting IL-17, but on the other hand, they stimulate osteoblasts to express RANKL to accelerate bone resorption [22, 23]. B cells, meanwhile, act as bone protectors by supplying about 40-60% of the total amount of osteoprotegerin, which inhibits osteoclast overactivation [24]. Together, these findings reveal that osteoporosis is not only an aging-related bone disease, but also a disease of immune-inflammation-mediated imbalance in skeletal homeostasis [25].

The reproductive system also plays a crucial role in osteoporosis. Oestrogen exerts a significant influence on the development of osteoporosis. When oestrogen levels decline, it promotes calcitonin-induced hypocalcaemia and a secondary increase in PTH levels, consistent with the mechanism of action of the RANK/RANK-L/OPG pathway [26]. There is increasing evidence showing the relationship between the digestive system and OP Inflammatory bowel disease: Crohn’s disease and ulcerative colitis will lead to malnutrition or impaired absorption of nutrients, calcium, and vitamin D deficiency, sex hormone deficiency, and even a dysbiotic intestinal microbiome. These factors can influence the onset and progression of OP [27, 28]. The nervous system is also involved, as pain signals are generated during bone absorption. In addition, the brain can influence the bones through the sympathetic nervous system. This pathway begins in the hypothalamus. After receiving signals such as leptin, the hypothalamus activates the sympathetic nervous system, which releases cortisol. This then acts on the β2-adrenergic receptor in the bone cells, reducing the amount of bone formation [29]. While COPD is found to be associated with OP, the mechanism underlying this association has been gradually unveiled between the respiratory system and OP, namely, low levels of vitamin D and calcium [30]. Finally, when it comes to the Urinary System, progressive kidney disease can lead to disturbances in calcium and phosphorus metabolism, followed by hyperparathyroidism, which eventually leads to abnormalities in bone structure and bone turnover Table **1** [31, 32].

## 4 ANTI-OP EFFECTS OF HERBS FUNCTIONING ON DIFFERENT SYSTEMS

Traditional Chinese medicine encompasses thousands of herbs with diverse therapeutic effects. Among these, we selected several herbs that have demonstrated efficacy against OP by acting on multiple physiological systems.

**Herba epimedii (*Epimedium brevicornu, Berberidaceae*)** is a valuable Chinese herb, and its extract Icariin is a flavonoid. It has been shown to have anti-OP effects, not only activating BMSCs through the BMP signalling pathway, Wnt/β-catenin pathway, JNK, and reactive oxygen species (ROS) levels, but also promoting osteogenesis through the estrogen receptor (ER) and extracellular signal-regulated kinase (ERK) signalling pathways, the Wnt signalling pathway, and the RANKL level [33-37]. As for osteoclasts, Icariin inhibited the inflammatory signaling pathways, like p38, ERK, nuclear factor kappa B(NF-κB), and JNK [38-40]. Nowadays, Icariin also plays a vital role in the prevention and treatment of cardiovascular diseases(CVDs) and neurological diseases like neurodegeneration, chronic inflammation, cognitive loss, depression, and diabetes [41-47]. It affects human monocytes, thereby reducing the inflammatory response. As for the immune system, it mainly influenced MAPK and NF-κB and promoted glucocorticoid receptors (GR), Nuclear factor erythroid 2-related factor 2 (Nrf2), and PI3K/AKT [48, 49]. What’s more, Icariin even plays a role in digest system by regulating the Gut Microbiota to ameliorate OP [50-52].

**Cistanches Herba (*Cistanche deserticola, Orobanchaceae*)** is regarded as the ginseng in the desert. Modern pharmacological studies have found that the main components of Cistanchis are: phenyl glycosides, cyclic enol ether terpenes and their glycosides, lignans and their glycosides, monoterpene glycosides, alkaloids, and saccharides [53]. It has a variety of biological activities such as anti-aging, liver protection, relief of physical fatigue, anti-osteoporosis, and laxative [54]. Cistanchis can directly or indirectly affect osteoblast and osteoclast activities by increasing the level of sex hormones, regulating the balance of calcium and phosphorus metabolism, antioxidant effects, influencing cytokines, and supplementing mineral elements, thus playing a role in the prevention and treatment of OP. Cis A is one of the main active components of Cistanches and is classified as a phenylethanol glycoside. It has anti-inflammatory and antioxidant activities [55]. It was investigated that Cis A could inhibit radiation-induced apoptosis, oxidative stress, and release of inflammatory factors in mouse lung tissues by inhibiting the activation of the TGF-β1/Vascular Endothelial Growth Factor(VEGF) signalling pathway and thereby inhibiting radiation-induced apoptosis [56]. In the field of orthopaedic research, Cis A was found to significantly increase bone mineralisation in de-ovulated mice, coordinating NF-κB inhibition and stimulation of the PI3K/AKT pathway to promote bone formation and reduce bone resorption. Other studies have demonstrated that Cis A can affect the RANKL/RANK/TRAF6 pathway *via* autophagy and thus bone resorption involving osteoclasts [57, 58].

**Eucommiae Cortex (*Eucommia ulmoides, Eucommiaceae*)**, a typical Chinese herb rich in numerous chemical constituents, consists of flavonoids, iridoids, phenolics, steroids, terpenoids, and lignans. It not only possesses anti-OP capabilities but also exhibits potential function in regulating different diseases, such as hypertension, hyperglycemia, diabetes, obesity, sexual dysfunction, and Alzheimer's disease, which range from the endocrine system, nervous system, to reproductive system [59, 60]. Evidence proves that Eucommia extract may prevent OP by restoring serum calcium, alkaline phosphatase, and osteocalcin levels, whilst regulating the serum OPG/RANKL ratio to within the normal range [61]. Multiple bioactive constituents underlie these effects *via* distinct mechanisms. Total lignans from Eucommia ulmoides (TL) have been shown to promote bone formation in osteoblasts and inhibit osteoclast activity, primarily by upregulating OPG expression and downregulating RANKL expression [62]. 5-(hydroxymethyl)-2-furaldehyde (5-HMF), reduced the demonstration of adipogenesis-markers (PPARγ, FABP4, C/EBPα, and LPL) of BMSCs in adipogenic induction medium [63]. Quercetin protected osteoblasts while inhibiting bone resorption of osteoclasts at the same time, as well as protected against iron overload-induced osteoporosis *via* the Nrf2/HO-1 pathway [64, 65]. Besides, recent studies have demonstrated that Eucommia ulmoides alters lipid metabolism by controlling the gut microbiota, which, in turn, will influence the OP [66].

**Psoraleae Fructus (*Psoralea corylifolia, Fabaceae*)** has a wide range of pharmacological effects, including antibacterial, antitumour, antiviral, antioxidant, anti-OP, increasing skin pigmentation and enhancing the immune system, increasing skin pigmentation and enhancing immune function. As it has a significant effect on ERα and ERβ, it mainly activated osteoblasts by Wnt/β-catenin pathways, BMP, MAPK, and IRE1 signaling, while inhibiting osteoclasts by AKT and AP-1 pathway [67-69].

**Rehmanniae Radix (*Rehmannia glutinosa, Orobanchaceae*)**, a sweet-tasting herb with both medicinal and edible properties, is known in Chinese as Dihuang. Research indicates that the compounds of Rehmanniae Radix are also widely distributed in the kidney, liver, heart, spleen, and lung, suggesting its potential to influence multiple systems. Experiments on osteoporotic rats have shown that this herbal medicine can improve OP. However, it does not exert its effects by directly supplementing oestrogen, but rather by regulating the body’s own steroid hormone metabolic network, thereby inhibiting bone loss [70, 71].

**Angelicae Sinensis Radix (*Angelica sinensis, Apiaceae*)** is a frequently used conventional Chinese medicine. The effects of Angelica sinensis have been elucidated in a variety of diseases, such as blood circulation, anti-aging, anti-tumour, and anti-inflammatory effects, and the latest study shows that Angelica polysaccharide promotes proliferation and osteoblast differentiation of BMSCs by regulation of long non-coding RNA H19 [72].

**Drynariae Rhizoma (*Drynaria fortunei, Polypodiaceae*),** traditionally used for treating wounds and bone fractures, has subsequently been employed as a potent herb for OP. The latest study shows it enhances bone calcification *via* activating osteoblast activity and suppressing bone resorption [73].

## 5 ANTI-OP EFFECTS OF FORMULA FUNCTIONING ON DIFFERENT SYSTEMS

**GuShuKang(GSK)** is a herbal Chinese formula, made up of seven herbs including Rhizoma Drynariae, Herba Epimedii, Rehmannia glutinosa, and Radix Astragali, *etc * [74]. Evidence shows that it can reduce bone loss and enhance BMD in patients of OP as well as inhibiting apoptosis in OVX rats through BMP-2/Smads signaling pathway [75, 76]. Recently, GSK was found to play a role in the endocrine and urinary System to anti-OP, which mainly demonstrated GSK regulated calcium metabolism through calciotropic hormones in serum and calcium transporters in duodenum and kidney of aged mice [74].

**Qing’E Formula (QEF)** consists of four Chinese medicinal herbs: Eucommiae Cortex, Psoraleae Fructus, Juglandis Semen, and Garlic Rhizoma. Clinically, it has been widely employed in postmenopausal women for the management of OP, chloasma, and CVDs [77]. And it has been elucidated that QEF showed the greatest estrogen-like effects compared to the individual components, indicating the synergistic mechanism of the herbal components.

**Erxian decoction (EXD)** is a typical traditional Chinese herbal formula for menopausal syndromes [78]. Curculiginis Rhizome, Epimedii Folium, Morindae Officinalis Radix, Angelicae Sinensis Radix, Anemarrhenae Rhizoma, Phellodendri Chinensis Cortex are its main components. At first, the function of EXD in OP was its estrogen-like osteoprotective effects [79]. Later, they found Nrf2/NF-κB signaling pathway is vital in anti-OP [80]. Recently, researchers found that Er-Xian decoction attenuates ovariectomy-induced osteoporosis by modulating fatty acid metabolism and the IGF1/PI3K/AKT signaling pathway [81].

**Zhuanggu Busui Formula (ZGBSF)** is a TCM formula including Epimedium, Dipsaci Radix, Paeoniae Radix Alba, Chuanxiong Rhizoma, Hedysarum Multijugum Maxim, Rehmanniae Radix, Praeparata, Eucommiae Cortex, Achyranthis Bidentatae Radix, Carthami Flos, Angelicae Sinensis Radix. ZGBSF is widely used in clinical practice and has potent anti-OP power. Studies showed that ZGBSF decreased the apoptosis rate in osteoblasts and promoted osteogenesis by the PI3K pathway [82].

**You-Gui-Yin** is a widely used TCM formula composed of multiple herbs, including Persicae Semen, Eucommiae Cortex, Cinnamomi Cortex, Monkshood Root, Lycii Fructus, Carthami Flos, Dioscoreae Rhizome, Glycyrrhizae Radix, Corni Fructus, and Rehmanniae Radix Preparata. It has been reported that You-Gui-Yin is involved in some chronic diseases, such as osteoarthritis [83, 84]. A recent study demonstrates that the mechanism by which You-Gui-Yin alleviates bone loss in Postmenopausal Osteoporosis(PMOP) involves the inhibition of NF-κB signaling, thereby preventing osteoclast formation [85].

**Bu-Shen-Tong-Luo decoction (BSTLD)** is composed of Epimedii folium, Drynariae rhizoma, Dipsaci radix, Poria, Paeoniae radix alba, Scolopendra, Scorpio, and Glycyrrhizae radix et rhizoma. It exerts a dual effect in animal models by stabilizing Hypoxia-Inducible factor 1-alpha(HIF-1α) through CTSK inhibition: it upregulates VEGF expression to promote angiogenesis while modulating the RANKL/OPG signaling axis to influence bone resorption processes [86].

**QiangGuYin (QGY)** is a commonly used TCM formula for the treatment of PMOP. It is composed of Lonicerae Japonicae Caulis, Cornu Cervi Degelatinatum, Spatholobi Caulis, Nidus Vespae, *etc*. Research indicates that QGY treatment significantly improves bone quality in OP rats, with its mechanism involving the regulation of multiple bone-metabolism signaling pathways. This formulation exerts therapeutic effects by inhibiting bone resorption by downregulating CKIP-1, LC3II/I, and RANKL, while simultaneously promoting bone formation by upregulating p62, p-AKT/AKT, p-mTOR/mTOR, RUNX2, and OPG [87].

## 6 DISCUSSION

This paper systematically reviews the latest research advances on how single Chinese herbal medicines and compound formulas prevent and treat OP through multi-system, multi-target pathways. Studies indicate that various Chinese herbs and classic formulae not only exert effects by regulating classic bone metabolism signaling pathways such as Wnt/β-catenin, BMP/Smad, and OPG/RANK/RANKL pathways, but also indirectly improve bone metabolism through multiple pathways, such as modulating gut microbiota, suppressing chronic inflammation, optimizing sex hormone levels, and regulating neuroendocrine functions. Notably, recent Western medical research has begun to show similar trends, gradually recognizing the close connections between other tissues like the lungs and liver and the skeletal system.

TCM emphasizes “differentiating syndromes to identify causes and treating based on thorough analysis of causes.” Therefore, the synergistic effects between herbs in Chinese medicinal formulas are particularly crucial. This article examines the pathogenesis of OP from a multisystem perspective, lists commonly used Chinese herbs and formulas for treating OP, and explains their regulatory roles across multiple systems (Table **2**). Current Western pharmaceutical treatments for OP primarily focus on single-target drugs that inhibit bone resorption or promote bone formation. While effective to some extent, long-term use may lead to adverse reactions and often neglects the body's holistic regulation. In contrast, TCM, through its multi-component, multi-pathway, and multi-target characteristics, not only regulates bone metabolism but also improves the functional state of related systems, demonstrating unique therapeutic advantages. Nevertheless, current research faces several limitations: most studies remain at the basic research stage, lacking high-quality clinical evidence; the complex chemical composition of TCM formulas means their active component groups and interaction mechanisms are not fully elucidated; and network pharmacology studies on TCM's multi-system regulation require deeper experimental validation. Based on the above analysis, future research should focus on the following directions:

- a.Utilize systems biology and network pharmacology methods to construct multidimensional interaction networks linking “constituents-targets-pathways-systems” in TCM, thereby deeply elucidating the scientific basis of its multisystem regulation.
- Strengthen clinical research by conducting multicenter randomized controlled trials to validate the clinical efficacy and safety of TCM in preventing and treating osteoporosis.
- Deepen research into the mechanisms of TCM regulation in emerging fields such as the “gut-bone axis,” “nerve-bone axis,” and “immune-bone axis.”
- Strengthen standardized quality research on TCM to ensure consistent quality of herbal materials and formulations.

## CONCLUSION

The onset and progression of OP involve dysfunction across multiple systemic functions rather than isolated skeletal lesions. Traditional perspectives primarily focused on bone metabolism itself or endocrine factors. However, modern research indicates that the immune, digestive, nervous, respiratory, and urinary systems are closely linked in maintaining bone homeostasis. This perspective of multisystem interactions aligns closely with TCM concepts such as the “holistic view” and theories like “the kidney governs bones, the spleen governs muscles, and the liver governs tendons.” Treatment thus focuses not only on the bones themselves but also on harmonizing the entire body's condition. With continuous advancements in modern research methodologies, TCM will undoubtedly provide more effective treatment options for osteoporosis prevention and treatment. Integrated treatment strategies combining Chinese and Western medicine may represent a key future direction in osteoporosis prevention and treatment, embodying the fusion and innovation of TCM's “holistic concept” with modern medicine's systems biology approach.

## Figures and Tables

**Table 1:** Association of different systems with osteoporosis.

**System**	**Association Mechanism**	**Key Factors**	**Reference Number**
Endocrine System	Core regulator of bone metabolism. Hormonal, glucose, and lipid balance directly influence osteoblastic /osteoclastic activity.	PTH, glucose, lipid metabolism	[17-19]
Immune System	Chronic inflammation promotes bone resorption. Inflammatory cytokines stimulate osteoclast formation.	TNF-α,IL-6,IL-17,T cells,B cells	[20-25]
Reproductive System	Declining estrogen accelerates bone loss, disrupting calcium balance and the RANKL/OPG system.	Estrogen, RANKL/OPG	[26]
Digestive System	Intestinal diseases cause malabsorption and dysbiosis, indirectly triggering osteoporosis.	IBD, calcium/vitamin D deficiency	[27,28]
Nervous System	The hypothalamus inhibits bone formation *via* the leptin-sympathetic pathway. Bone resorption triggers pain.	Leptin, Norepinephrine, Adrβ2 Receptor	[29]
Respiratory System	Osteoporosis is highly prevalent in pulmonary disease patients, associated with Vitamin D deficiency and hypocalcemia.	COPD, Low Vitamin D	[30]
Urinary System	Kidney disease causes calcium-phosphorus metabolism disorders and hyperparathyroidism, directly damaging bones.	CKD, Secondary Hyperparathyroidism	[31,32]

**Table 2:** Summary of multisystem effects and mechanisms of single-herb and compound formulations in traditional chinese medicine for osteoporosis treatment.

**Name**	**Type**	**Key Active Ingredient/Composition**	**Core Anti-OP Mechanism**	**Effects on Other Systems**
Herba Epimedii	Single Agent	Icariin	**Promotes Bone Formation**: Activates BMP, Wnt/β-catenin, and ERK signaling pathways as well as the ER in BMSCs and osteoblasts [33-37]. **Inhibits Bone Resorption**: Suppresses osteoclast activity by inhibiting the NF-κB, p38, and JNK pathways [38-40]. **Modulates Bone Microenvironment**: Regulates the RANKL/OPG axis and activates the Nrf2 antioxidant pathway [33-37, 48, 49].	Cardiovascular, neuroprotective, immunomodulatory, and gut microbiota-modulating effects [41-52].
Cistanches Herba	Single Agent	Cistanchin (Cis A)	**Promotes Bone Formation**: Activates the PI3K/AKT pathway to enhance osteoblast function [57,58]. **Inhibits Bone Resorption**: Intervenes in the RANKL/RANK/TRAF6 pathway and autophagy to suppress osteoclastogenesis and activity [57, 58]. **Modulates Bone Microenvironment**: Improves the skeletal metabolic milieu through multi-pathway regulation of sex hormones, calcium-phosphorus metabolism, and antioxidant effects [54].	Anti-aging, hepatoprotective, anti-fatigue, laxative, anti-inflammatory, and antioxidant effects; inhibits radiation-induced lung injury [54-56].
Eucommiae Cortex	Single Agent	Flavonoids (*e.g.*, Quercetin), Total Lignans, Iridoids, Phenolics (*e.g.*, 5-HMF)	**Promotes Bone Formation**: Restores serum osteocalcin levels; TL upregulates OPG expression in osteoblasts [61, 62]. **Inhibits Bone Resorption**: Regulates serum OPG/RANKL ratio; TL downregulates RANKL expression; Quercetin inhibits osteoclast activity [61, 62, 64, 65]. **Modulates Bone Microenvironment**: 5-HMF inhibits BMSC adipogenesis; modulates lipid metabolism and gut microbiota [63, 66].	Potential therapeutic effects on hypertension, diabetes, obesity, sexual dysfunction, and Alzheimer's disease [59, 60].
Psoraleae Fructus	Single Agent	Psoralen	**Promotes Bone Formation**: Activates osteoblasts *via* Wnt/β-catenin, BMP, MAPK, and IRE1 signaling pathways, with significant effects on estrogen receptors (ERα/ERβ) [67-69]. **Inhibits Bone Resorption**: Suppresses osteoclast activity through the AKT and AP-1 pathways [67-69].	Antibacterial, antitumor, antiviral, antioxidant properties, and enhances immune function.
Rehmanniae Radix	Single Agent	-	**Promotes Bone Formation**: Regulates steroid hormone metabolic network to promote bone formation [70-71]. **Modulates Bone Microenvironment**: Inhibits bone loss by modulating endogenous steroid hormone metabolism, without directly supplementing estrogen [70, 71].	Compounds distribute to the kidney, liver, heart, spleen, and lung, indicating broad systemic regulatory potential.
Angelica sinensis	Single Agent	Angelica Polysaccharide	**Promotes Bone Formation**: Stimulates BMSC proliferation and osteogenic differentiation *via* lncRNA H19 regulation [72].	Promotes blood circulation and exerts anti-aging, anti-tumor, and anti-inflammatory activities [72].
Drynariae Rhizoma	Single Agent	-	**Promotes Bone Formation**: Activates osteoblast activity [73]. **Inhibits Bone Resorption**: Suppresses bone resorption [73].	Traditionally used to treat wounds and bone fractures.
GSK	Formula Functioning	*Dipsacus asper*, Epimedium, Rehmannia, Astragalus, *etc*	**Promotes Bone Formation**: Inhibits osteoblast apoptosis *via* BMP-2/Smads pathway [75,76]. **Modulates Bone Microenvironment**: Regulates calcium homeostasis *via* hormones and intestinal/renal transporters [74].	Exerts effects on the endocrine and urinary systems [74].
Qing’ E Formula (QEF)	Formula Functioning	Eucommia bark, *Psoralea corylifolia*, Walnut kernel, Garlic	**Promotes Bone Formation**: Synergistic estrogen-like effects [77]. **Inhibits Bone Resorption**: Likely *via* estrogenic action[77]. **Modulates Bone Environment**: Improves postmenopausal metabolic milieu [77].	Manages chloasma and CVDs [77].
EXD	Formula Functioning	Cynomorium, Epimedium, Morinda, Angelica, Anemarrhena, Phellodendron	**Promotes Bone Formation**: Activates the IGF1/PI3K/AKT signaling pathway [81]. **Inhibits Bone Resorption**: Modulates the Nrf2/NF-κB signaling pathway [80]. **Modulates Bone Environment**: Attenuates osteoporosis by modulating fatty acid metabolism [81].	A typical formula for treating menopausal syndrome [78].
ZGBSF	formula functioning	Epimedium, Dipsacus, Rehmannia glutinosa, *etc*.	**Promotes Bone Formation**: Promotes osteogenesis and decreases osteoblast apoptosis *via* the PI3K/AKT pathway [82].	-
You-Gui-Yin	Formula Functioning	Aconite, Cinnamon, Eucommia, *etc*.	**Inhibits Bone Resorption**: Prevents osteoclast formation by inhibiting the NF-κB signaling pathway [85].	Involved in managing chronic diseases such as osteoarthritis [83,84].
BSTLD	Formula Functioning	Epimedii Folium, Drynariae Rhizoma, *etc*	**Promotes Bone Formation**: Upregulates VEGF to promote angiogenesis [86]. **Inhibits Bone Resorption**: Modulates RANKL/OPG axis [86]. **Modulates Bone Environment**: Stabilizes HIF-1α *via* CTSK inhibition [86].	-
QiangGuYin	Formula Functioning	*Lonicerae japonicae* Caulis, Spatholobi Caulis, Nidus Vespae, *etc*.	**Promotes Bone Formation**: Upregulates p-AKT/AKT, p-mTOR/mTOR, RUNX2, and OPG to stimulate osteogenesis [87]. **Inhibits Bone Resorption**: Downregulates CKIP-1, LC3II/I, and RANKL to suppress osteoclast activity [87]. **Modulates Bone Environment**: Regulates autophagy flux (p62, LC3II/I) to remodel the bone metabolic microenvironment [87].	-

## LIST OF ABBREVIATIONS

ALP: Alkaline Phosphatase
BMD: Bone Mineral Density
BMP: Bone Morphogenetic Protein
BMSCs: Bone Marrow Mesenchymal Stem Cells
CVDs: Cardiovascular Diseases
ER: Estrogen Receptor
ERK: Extracellular Signal-Regulated Kinase
GLP-1: Glucagon-Like Peptide-1
HIF-1α: Hypoxia-Inducible Factor 1-Alpha
IBD: Inflammatory Bowel Disease
IGF: Insulin-Like Growth Factor
JNK: c-Jun N-Terminal Kinase
MAPK: Mitogen-Activated Protein Kinase
NF-κB: Nuclear Factor Kappa B
Nrf2: Nuclear Factor Erythroid 2-Related Factor 2
OP: Osteoporosis
OPG: Osteoprotegerin
PI3K: Phosphoinositide 3-Kinase
PTH: Parathyroid Hormone
RANK: Receptor Activator of Nuclear Factor Kappa-B
RANKL: Receptor Activator of Nuclear Factor Kappa-B Ligand
RUNX2: Runt-Related Transcription Factor 2
Smad: Mothers Against Decapentaplegic Homolog
TCM: Traditional Chinese Medicine
TNF-α: Tumor Necrosis Factor-Alpha
VEGF: Vascular Endothelial Growth Factor
